# Benefits of Icosapent Ethyl Across the Range of Kidney Function in Patients With Established Cardiovascular Disease or Diabetes: REDUCE-IT RENAL

**DOI:** 10.1161/CIRCULATIONAHA.121.055560

**Published:** 2021-10-28

**Authors:** Arjun Majithia, Deepak L. Bhatt, Allon N. Friedman, Michael Miller, Ph. Gabriel Steg, Eliot A. Brinton, Terry A. Jacobson, Steven B. Ketchum, Rebecca A. Juliano, Lixia Jiao, Ralph T. Doyle, Craig Granowitz, Matthew Budoff, R. Preston Mason, Jean-Claude Tardif, William E. Boden, Christie M. Ballantyne

**Affiliations:** Division of Cardiovascular Medicine, Lahey Hospital and Medical Center, Burlington, MA (A.M.).; Division of Cardiovascular Medicine, Brigham and Women’s Hospital, Harvard Medical School, Boston, MA (D.L.B., R.P.M.).; Department of Medicine, Indiana University School of Medicine, Indianapolis (A.N.F.).; Department of Medicine, University of Maryland School of Medicine, Baltimore (M.M.).; Université de Paris, FACT (French Alliance for Cardiovascular Trials), Assistance Publique–Hôpitaux de Paris, Hôpital Bichat, INSERM Unité 1148, France (P.G.S.).; Utah Lipid Center, Salt Lake City (E.A.B.).; Office of Health Promotion and Disease Prevention, Department of Medicine, Emory University School of Medicine, Atlanta, GA (T.A.J.).; Amarin Pharma, Inc., Bridgewater, NJ (S.B.K., R.A.J., L.J., R.T.D., C.G.).; Division of Cardiology, Harbor UCLA Medical Center, Torrance, CA (M.B.).; Montreal Heart Institute, Université de Montréal, Canada (J.-C.T.).; Division of Cardiovascular Medicine, Boston Medical Center, MA (W.E.B.).; Department of Medicine, Baylor College of Medicine, and Center for Cardiovascular Disease Prevention, Methodist DeBakey Heart and Vascular Center, Houston, TX (C.M.B.).

**Keywords:** eicosapentaenoic acid ethyl ester, fatty acids, fatty acids, omega-3, lipids, prevention and control, renal insufficiency, chronic, triglycerides

## Abstract

Chronic kidney disease is associated with adverse outcomes among patients with established cardiovascular disease (CVD) or diabetes. Commonly used medications to treat CVD are less effective among patients with reduced kidney function.

**Methods:**

REDUCE-IT (Reduction of Cardiovascular Events with Icosapent Ethyl-Intervention Trial) was a multicenter, double-blind, placebo-controlled trial that randomly assigned statin-treated patients with elevated triglycerides (135–499 mg/dL) who had CVD or diabetes and 1 additional risk factor to treatment with icosapent ethyl (4 g daily) or placebo. Patients from REDUCE-IT were categorized by prespecified estimated glomerular filtration rate (eGFR) categories to analyze the effect of icosapent ethyl on the primary end point (composite of cardiovascular death, nonfatal myocardial infarction, nonfatal stroke, coronary revascularization, or unstable angina) and key secondary end point (a composite of cardiovascular death, nonfatal myocardial infarction, or nonfatal stroke).

**Results:**

Among the 8179 REDUCE-IT patients, median baseline eGFR was 75 mL·min^–1^·1.73 m^–2^ (range, 17–123 mL·min^–1^·1.73 m^–2^). There were no meaningful changes in median eGFR for icosapent ethyl versus placebo across study visits. Treatment with icosapent ethyl led to consistent reduction in both the primary and key secondary composite end points across baseline eGFR categories. Patients with eGFR <60 mL·min^–1^·1.73 m^–2^ treated with icosapent ethyl had the largest absolute and similar relative risk reduction for the primary composite end point (icosapent ethyl versus placebo, 21.8% versus 28.9%; hazard ratio [HR], 0.71 [95% CI, 0.59–0.85]; *P*=0.0002) and key secondary composite end point (16.8% versus 22.5%; HR 0.71 [95% CI, 0.57–0.88]; *P*=0.001). The numeric reduction in cardiovascular death was greatest in the eGFR <60 mL·min^–1^·1.73 m^–2^ group (icosapent ethyl: 7.6%; placebo: 10.6%; HR, 0.70 [95% CI, 0.51–0.95]; *P*=0.02). Although patients with eGFR <60 mL·min^–1^·1.73 m^–2^ treated with icosapent ethyl had the highest numeric rates of atrial fibrillation/flutter (icosapent ethyl: 4.2%; placebo 3.0%; HR 1.42 [95% CI, 0.86–2.32]; *P*=0.17) and serious bleeding (icosapent ethyl: 5.4%; placebo 3.6%; HR, 1.40 [95% CI, 0.90–2.18]; *P*=0.13), HRs for atrial fibrillation/flutter and serious bleeding were similar across eGFR categories (*P*-interaction for atrial fibrillation/flutter=0.92; *P*-interaction for serious bleeding=0.76).

**Conclusions:**

In REDUCE-IT, icosapent ethyl reduced fatal and nonfatal ischemic events across the broad range of baseline eGFR categories.

**REGISTRATION:** URL: https://www.clinicaltrials.gov; Unique identifier: NCT01492361

## Abstract 2

Supplemental Digital Content is available in the text.

**Clinical Perspective**

**What Is New?**

- Icosapent ethyl reduced cardiovascular events among patients with elevated triglycerides and well-controlled low-density lipoprotein cholesterol on statin therapy across a wide range of baseline kidney function.

**What Are the Clinical Implications?**

- Despite having a well-controlled low-density lipoprotein cholesterol on statin therapy, patients with elevated triglycerides have significant residual risk for coronary events.
- Treatment with icosapent ethyl has been shown to significantly reduce cardiovascular events and mortality in this patient population.
- These findings are applicable to patients with chronic kidney disease across the spectrum of baseline kidney function.

Drug therapies that target low-density lipoprotein cholesterol in patients with established cardiovascular disease (CVD) or cardiovascular risk factors can improve survival, prevent first or subsequent cardiovascular events, and reduce the need for coronary revascularization.^1–4^ Although hypertriglyceridemia is an independent predictor of cardiovascular events, randomized studies of medications that lower triglyceride levels, including niacin and fibrates, have had less consistent success in improving cardiovascular outcomes.^5–9^

Contemporary studies of marine-derived long-chain polyunsaturated n-3 fatty acid mixtures, which can effectively lower triglyceride levels, have not demonstrated reductions in cardiovascular events among statin-treated patients.^10–13^ However, clinical benefit may differ based on the particular lipid composition of the n-3 fatty acid formulation. Icosapent ethyl contains the ethyl ester of a single long-chain murine omega-3 fatty acid, eicosapentaenoic acid. REDUCE-IT (Reduction of Cardiovascular Events with Icosapent Ethyl-Intervention Trial) randomly assigned 8179 statin-treated patients with established CVD or diabetes and other cardiovascular risk factors to either 4 g daily of icosapent ethyl or matching placebo.^14–20^ After a median follow-up period of 4.9 years, the study drug demonstrated a 25% relative risk reduction in the primary composite end point of cardiovascular death, myocardial infarction, stroke, coronary revascularization, or unstable angina. The study further demonstrated that patients treated with icosapent ethyl had a 26% relative risk reduction in the composite of cardiovascular death, myocardial infarction, or stroke.

Among patients with chronic kidney disease (CKD), CVD remains the leading cause of morbidity and mortality.^21–23^ However, because of the gaps in data and negative study results, uncertainty exists over the benefits of applying proven CVD therapy in the general population to the CKD population, especially in patients with advanced kidney disease.^24–27^ This analysis aimed to explore the effects of icosapent ethyl versus placebo across the range of kidney function among patients enrolled in the REDUCE-IT study.

## Methods

### Study Design and Patient Characteristics

The data that support the findings of this study may be made available from the corresponding author on reasonable request. The study design and main results of the REDUCE-IT trial have been published previously.

REDUCE-IT was an international, phase 3b, double-blind trial that randomly assigned patients to treatment with icosapent ethyl 4 g daily (2 g twice daily with food) or matching placebo. Verbal and written informed consent were obtained from all study participants, and all sites were approved by institutional review boards.

Patients met eligibility criteria for enrollment if they had established CVD (secondary prevention group) or if they had type 1 or type 2 diabetes on medical treatment, and if they were ≥50 years of age and had at least 1 other major cardiovascular risk factor (high-risk primary prevention group). All patients were required to be on statin therapy for at least 4 weeks and to have a low-density lipoprotein cholesterol level between 41 mg/dL and 100 mg/dL. In addition, study patients had baseline triglyceride levels between 135 mg/dL and 500 mg/dL. Key exclusion criteria included severe heart failure, planned coronary intervention or surgery, severe liver disease, hemoglobin A1c level >10%, a history of pancreatitis, or known hypersensitivity to fish, shellfish, or ingredients of icosapent ethyl or placebo.

### Measurement of Kidney Function

Estimated glomerular filtration rate (eGFR) was calculated using the Chronic Kidney Disease Epidemiology Collaboration equation as follows: eGFR=141×min (Scr/κ, 1)α×max(Scr/κ, 1)–1.209×0.993Age×1.018 [if female] × 1.159 [if Black], where Scr is serum creatinine in mg/dL, κ is 0.7 for women and 0.9 for men, α is –0.329 for women and –0.411 for men, min indicates the minimum of Scr/κ or 1, and max indicates the maximum of Scr/κ or 1.

### End Points and Follow-Up

The primary efficacy end point was a composite of cardiovascular death, nonfatal myocardial infarction, nonfatal stroke, coronary revascularization, or hospitalization for unstable angina. The key secondary end point was a composite of cardiovascular death, nonfatal myocardial infarction, or nonfatal stroke. In addition, the application of eGFR categories to prespecified hierarchical testing of individual and composite end points was performed. An independent committee blinded to treatment performed end point adjudication.

### Statistical Analysis

For this analysis, the effects of icosapent ethyl versus placebo were examined within prespecified estimated eGFR categories (eGFR <60 mL·min^–1^·1.73 m^–2^, 60 to <90 mL·min^–1^·1.73 m^–2^, ≥90 mL·min^–1^·1.73 m^–2^). As post hoc analysis, study patients were further classified by additional eGFR categories corresponding to CKD stage (>15 to <30 mL·min^–1^·1.73 m^–2^, ≥30 to <45 mL·min^–1^·1.73 m^–2^, ≥45 to <60 mL·min^–1^·1.73 m^–2^, ≥60 mL·min^–1^·1.73 m^–2^). Demographic and baseline characteristics were compared between treatment groups within each category. This analysis used the χ^2^ test for comparison of categorical variables and the Wilcoxon rank-sum test for comparison of continuous variables. All efficacy analyses were performed according to the intention-to-treat principle. A Kaplan-Meier analysis stratified by cardiovascular risk category, geographic region, and baseline ezetimibe use depicted time to first occurrences of the primary and the secondary efficacy end points in the prespecified testing hierarchy. Hazard ratios (HRs) and 95% CIs were determined from a corresponding stratified Cox proportional-hazards regression model. Heterogeneity of treatment effects among the eGFR categories was examined by testing the interaction term of treatment by eGFR category in the Cox regression model. Statistical testing was based on a 2-sided significance level of 0.05 without adjustment for multiple comparisons. Statistical analyses were performed using SAS software, version 9.4 (SAS Institute).

## Results

Among the 8179 REDUCE-IT patients, the median baseline eGFR was 75 mL·min^–1^·1.73 m^–2^ (range, 17–123 mL·min^–1^·1.73 m^–2^). Of the patients, 1816 (22.2%) had eGFR <60 mL·min^–1^·1.73 m^–2^, 4455 (54.5%) had eGFR 60 to <90 mL·min^–1^·1.73 m^–2^, and 1902 (23.3%) had eGFR ≥90 mL·min^–1^·1.73 m^–2^. Baseline characteristics of patients across prespecified eGFR categories are described in Table 1. The median age of patients with eGFR <60 mL·min^–1^·1.73 m^–2^ was higher than in other eGFR categories. In each category, the presence of established CVD (secondary prevention) accounted for the majority of enrolled patients. At baseline, median low-density lipoprotein cholesterol was similar among icosapent ethyl- and placebo-treated patients (74 versus 76 mg/dL). There were no significant between-group differences in baseline low-density lipoprotein cholesterol or triglyceride levels.

**Table 1.**
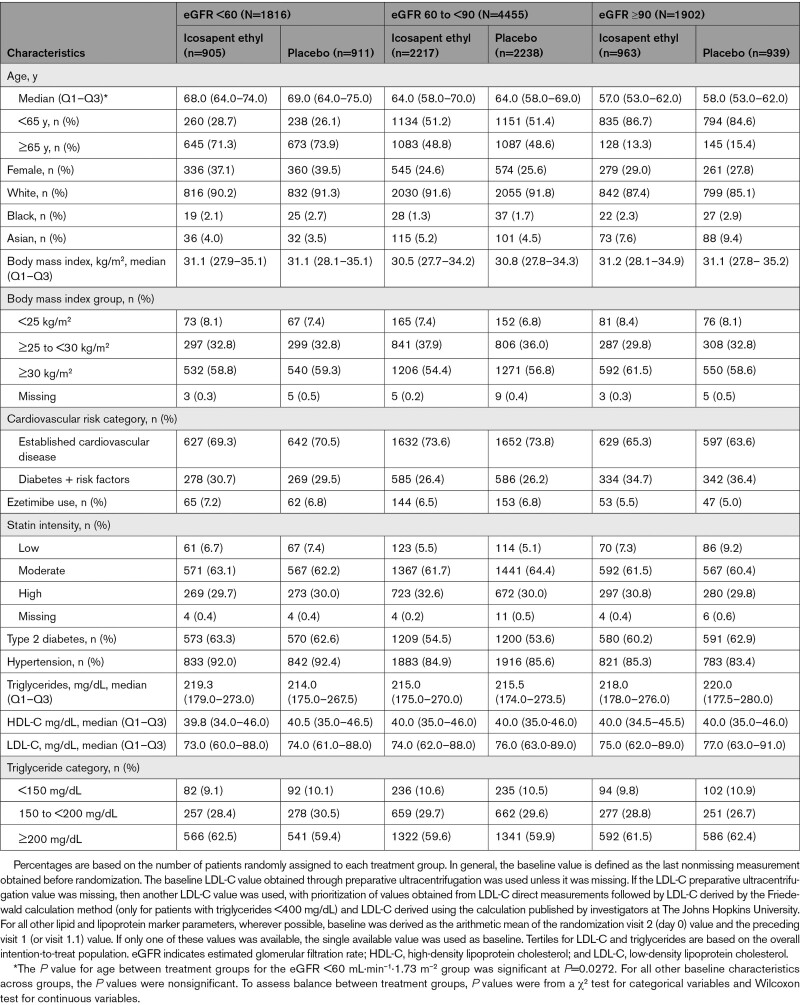
Key Baseline Characteristics, by Baseline eGFR (mL·min^–1^·1.73 m^–2^)

**Figure 1.**
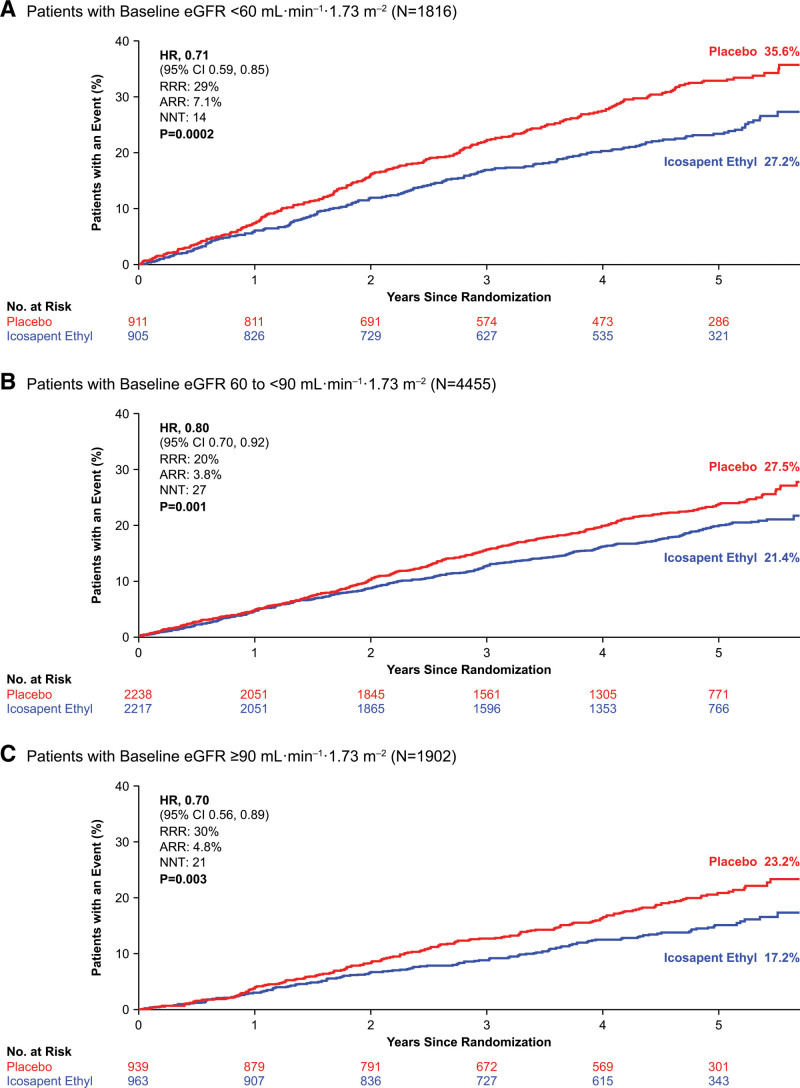
**Kaplan-Meier curves for the primary composite end point by eGFR subgroup.** **A**, Kaplan-Meier curves for the primary composite end point among patients with eGFR <60 mL·min^–1^·1.73 m^–2^. **B**, Kaplan-Meier curves for the primary composite end point among patients with eGFR 60 to <90 mL·min^–1^·1.73 m^–2^. **C**, Kaplan-Meier curves for the primary composite end point among patients with eGFR ≥90 mL·min^–1^·1.73 m^–2^. The *y* axis represents the cumulative incidence rate. Primary composite end point events were cardiovascular death, nonfatal myocardial infarction, nonfatal stroke, coronary revascularization, or hospitalization for unstable angina. Estimated Kaplan-Meier event rate at ≈5.7 years. The curves were visually truncated at 5.7 years because a limited number of events occurred beyond that time point; all patient data were included in the analyses. ARR indicates absolute risk reduction; eGFR, estimated glomerular filtration rate; HR, hazard ratio; NNT, number needed to treat; and RRR, relative risk reduction.

**Figure 2.**
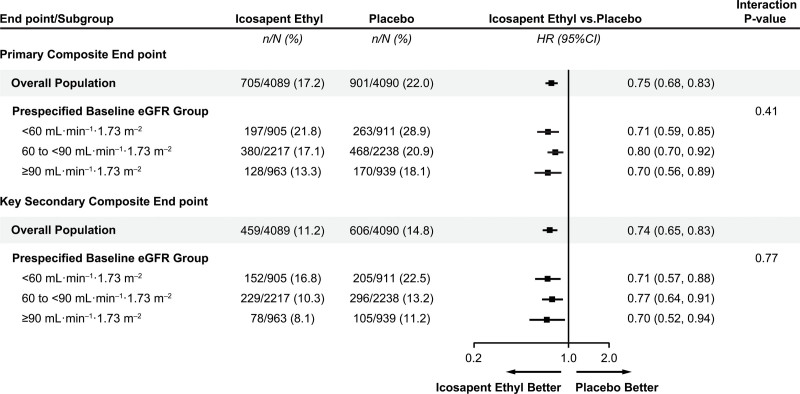
**Primary and key secondary composite end point event rates by eGFR category.** The primary composite end point and key secondary composite event rates by prespecified eGFR categories. eGFR indicates estimated glomerular filtration rate; and HR, hazard ratio.

At a median follow-up of 4.9 years, both the primary and secondary composite end points were significantly reduced among patients treated with icosapent ethyl. This therapy led to consistent reduction in both the primary and key secondary composite end points across baseline eGFR categories (Figure 1, Figure S1). Patients with eGFR <60 mL·min^–1^·1.73 m^–2^ treated with icosapent ethyl had the largest absolute and similar relative risk reduction for the primary composite end point (icosapent ethyl versus placebo, 21.8% versus 28.9%; HR, 0.71 [95% CI, 0.59–0.85]; *P*=0.0002) and key secondary composite end point (16.8% versus 22.5%; HR, 0.71 [95% CI, 0.57–0.88]; *P*=0.001; Figure 2, Figure S3). A post hoc analysis categorizing patients by commonly used eGFR categories corresponding to CKD stage also revealed a consistent reduction in primary and key secondary outcome event rates across eGFR categories of patients treated with icosapent ethyl (Figure S2).

Application of eGFR categories to prespecified hierarchical testing demonstrated consistent reductions, in general, in event rates across eGFR categories with icosapent ethyl as supported by nonsignificant interaction *P* values (Figure S3). Icosapent ethyl was associated with significant reductions in cardiovascular death or nonfatal myocardial infarction in eGFR <60 mL·min^–1^·1.73 m^–2^ (13.9% versus 18.3%; HR, 0.72 [95% CI, 0.57–0.91]; *P*=0.006) and eGFR 60 to <90 mL·min^–1^·1.73 m^–2^ (8.8% versus 11.4%; HR, 0.76 [95% CI, 0.63–0.92]; *P*=0.004) categories, with numeric reduction in the ≥90 mL·min^–1^·1.73 m^–2^ category. Similar differences were observed for fatal or nonfatal myocardial infarction and need for urgent or emergent revascularization.

The relative risk reductions in the primary composite and key secondary composite end points, in general, were consistent among icosapent ethyl–treated patients with either diabetes and risk factors for CVD (high-risk primary prevention cohort) or with established CVD (secondary prevention cohort) across eGFR subgroups (Figures S4 and S5). Event rates and absolute risk reductions were numerically highest among patients with eGFR <60 mL·min^–1^·1.73 m^–2^ in the established CVD cohort (23.6% versus 33.2%; *P*<0.0001). Among patients with diabetes and risk factors, higher event rates were observed in the lowest eGFR group, but a higher absolute risk reduction was not consistently observed.

In each of the eGFR categories, we observed consistent risk reductions in the primary and key secondary end points among icosapent ethyl–treated patients with triglyceride levels ≥200 mg/dL or <200 mg/dL (Figure S6).

A safety profile similar to the full cohort was observed for icosapent ethyl compared with placebo across eGFR subgroups. Adverse event rates rose with decreasing eGFR, but total adverse events occurred at similar rates with icosapent ethyl versus placebo.

Among icosapent ethyl–treated patients with eGFR <60 mL·min^–1^·1.73 m^–2^, there was a higher rate of bleeding-related disorders (18.0% versus 13.3%; *P*=0.007). The highest rate of serious bleeding events was observed in the eGFR <60 mL·min^–1^·1.73 m^–2^ category (5.4% versus 3.6%; *P*=0.07; Table 2). However, HRs for all bleeding and serious bleeding events were similar regardless of eGFR cutoff, with no significant interaction observed (*P* interactions for all bleeding=0.68 and serious bleeding=0.76; Figure S7). No significant differences in gastrointestinal or central nervous system bleeding events were observed between icosapent ethyl and placebo across eGFR categories. In addition, no significant differences in serious bleeding-related adverse events between icosapent ethyl and placebo across eGFR categories were observed (Tables 2–4).

**Table 2.**
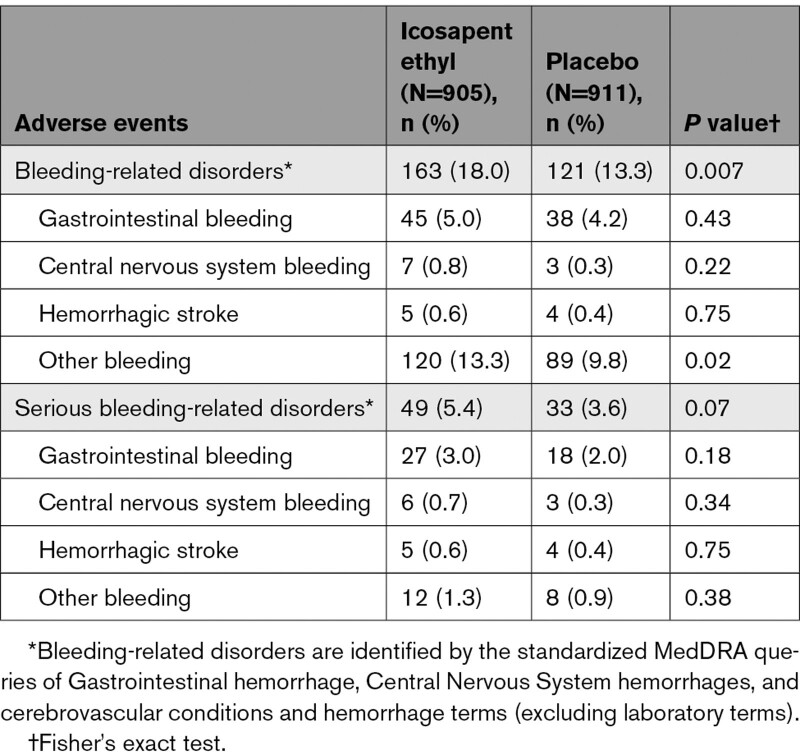
Summary of Bleeding-Related Adverse Events, by eGFR <60 mL·min^–1^·1.73 m^–2^

**Table 3.**
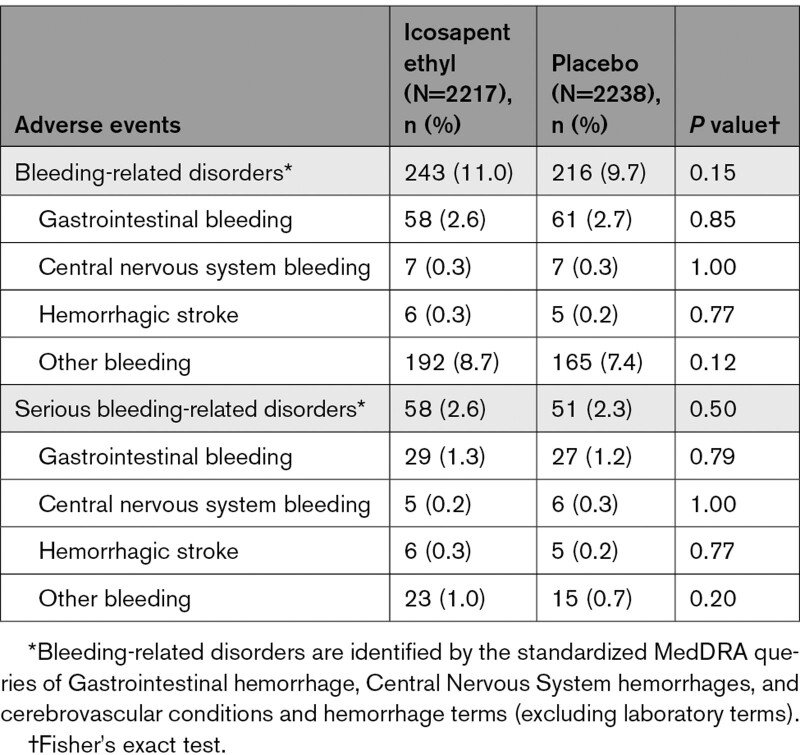
Summary of Bleeding-Related Adverse Events, by eGFR 60 to <90 mL·min^–1^·1.73 m^–2^

**Table 4.**
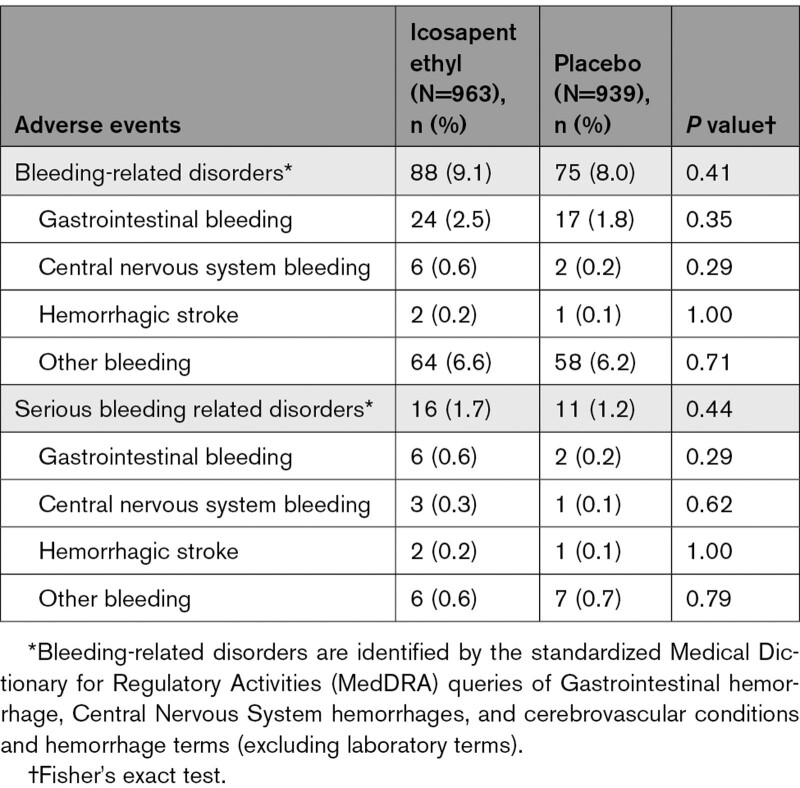
Summary of Bleeding-Related Adverse Events, by eGFR ≥90 mL·min^–1^·1.73 m^–2^

In each eGFR category, no significant difference was observed in rates of treatment emergent adverse events of atrial fibrillation or atrial flutter (Tables 5–7, Figure S7), although a significant difference had been noted in the trial overall. Atrial fibrillation/flutter requiring hospitalization was an adjudicated end point, and rates of positively adjudicated atrial fibrillation/flutter were higher in the eGFR <60 mL·min^–1^·1.73 m^–2^ subgroup, although absolute risk differences were similar across eGFR categories (icosapent ethyl: 4.2%; placebo 3.0%; HR, 1.42 [95% CI, 0.86–2.32]; *P*=0.17; Figure S7). The relative risk for atrial fibrillation among icosapent ethyl–treated patients was similar among eGFR categories, with no significant interaction observed (*P*-interaction=0.92; Figure S7).

**Table 5.**
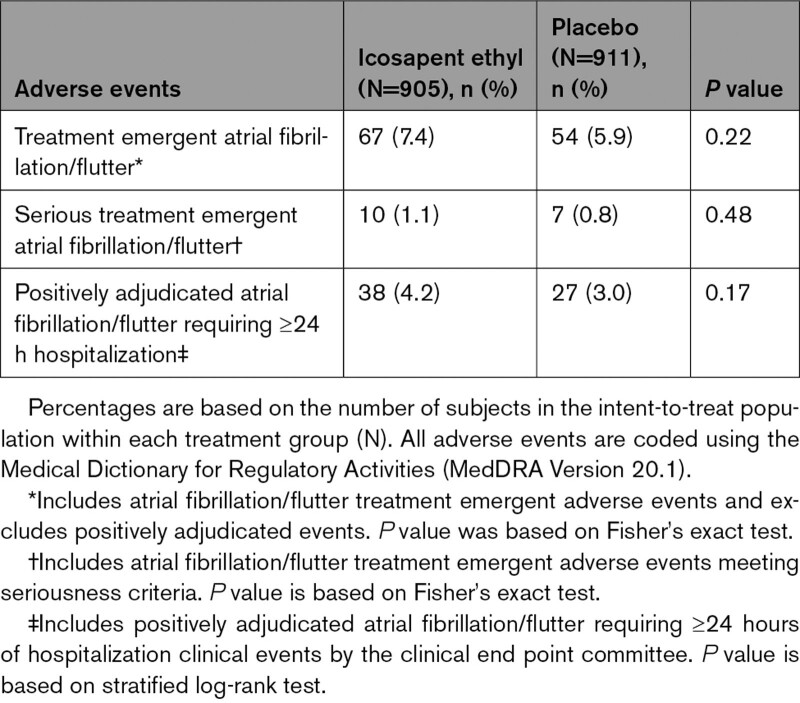
Summary of Atrial Fibrillation and Atrial Flutter Adverse Events and Positively Adjudicated Events, by eGFR <60 mL·min^–1^·1.73 m^–2^

**Table 6.**
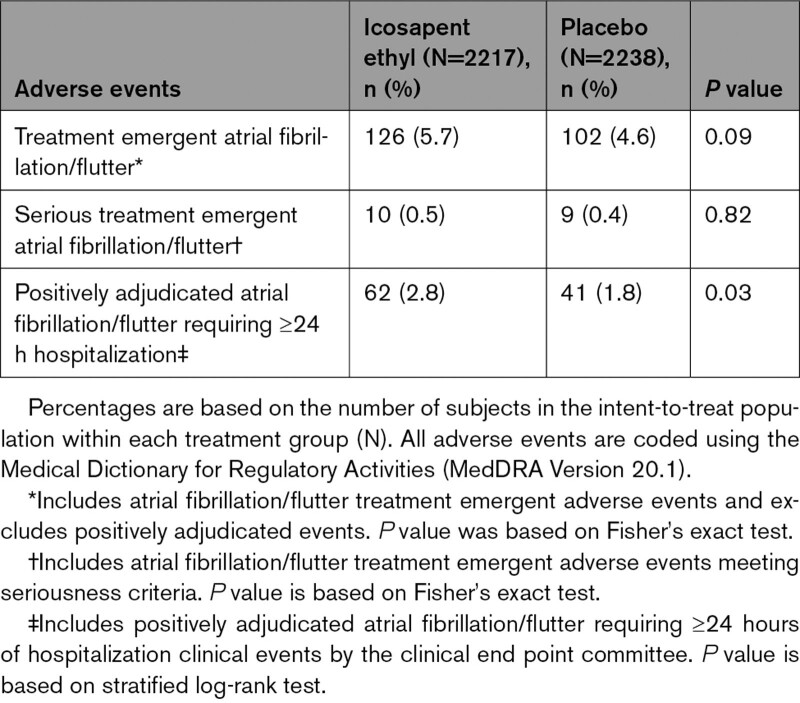
Summary of Atrial Fibrillation and Atrial Flutter Adverse Events and Positively Adjudicated Events, by eGFR 60 to <90 mL·min^–1^·1.73 m^–2^

**Table 7.**
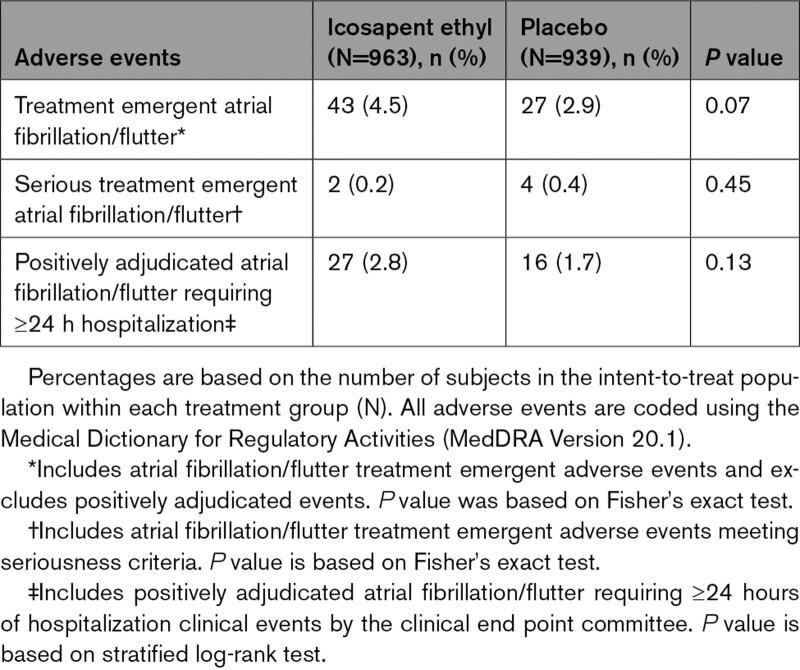
Summary of Atrial Fibrillation and Atrial Flutter Adverse Events and Positively Adjudicated Events, by eGFR ≥90 mL·min^–1^·1.73 m^–2^

There were no significant differences between icosapent ethyl and placebo in overall or serious treatment emergent adverse events (Table S1). Severe treatment emergent adverse events occurred more commonly among patients with eGFR <60 mL·min^–1^·1.73 m^–2^ but did not differ between icosapent ethyl and placebo. Microalbuminuria was reported at low rates, more commonly with placebo (14 versus 3; *P*=0.01), and expressed no clear trend across eGFR subgroup.

## Discussion

The REDUCE-IT study demonstrated a 25% reduction in the risk of the primary composite end point of cardiovascular death, nonfatal myocardial infarction, nonfatal stroke, coronary revascularization, or unstable angina among statin-treated patients randomly assigned to 4 g of icosapent ethyl (2 g twice daily) compared with those who were randomly assigned to placebo. This corresponds to a number needed to treat of 21 to prevent a cardiovascular event. In the current prespecified and post hoc analyses, we found that icosapent ethyl consistently reduced cardiovascular events across the full spectrum of baseline renal function categories in the REDUCE-IT study. Subgroup analyses demonstrated consistent reductions in the composite primary and key secondary end points for patients with eGFR <60 mL·min^–1^·1.73 m^–2^, 60 to <90 mL·min^–1^·1.73 m^–2^, and ≥90 mL·min^–1^·1.73 m^–2^. The greatest absolute reduction in composite primary and key secondary event rates was seen among patients with eGFR <60 mL·min^–1^·1.73 m^–2^. Among patients treated with icosapent ethyl with eGFR <60 mL·min^–1^·1.73 m^–2^, there was a 29% relative and 7.1% absolute reduction in the primary composite end point, corresponding to a number needed to treat of 14. Significant reductions in cardiovascular mortality or nonfatal myocardial infarction occurred among patients in the icosapent ethyl group with eGFR <60 mL·min^–1^·1.73 m^–2^ and 60 to <90 mL·min^–1^·1.73 m^–2^. We additionally observed numeric reductions in patients on this treatment with eGFR ≥90 mL·min^–1^·1.73 m^–2^.

Tolerability and safety remained consistent within the entire study cohort. Adverse events occurred at similar rates with icosapent ethyl and placebo but more frequently among patients with lower eGFR. A safety profile similar to the full cohort was observed for icosapent ethyl compared with placebo across eGFR subgroups, including an increase in total bleeding events without an increase in serious central nervous system or gastrointestinal bleeding, and an increase in the rates of atrial fibrillation or atrial flutter. Overall, bleeding rates were higher with decreasing eGFR, but the relative risks for all and serious bleeding were similar across eGFR categories, with no significant interaction observed. Atrial fibrillation/flutter event rates were higher in the lowest eGFR group, although absolute and relative risk differences were similar to those observed in other eGFR subgroups with no significant interaction observed.

CKD is strongly associated with dyslipidemia, and CVD remains the leading cause of mortality among patients with CKD.^25,26,28^ Commonly used cardiovascular medications that treat dyslipidemia may be ineffective among patients with severe CKD. The benefit of statin-based therapy decreases as eGFR declines. Limited data exist on the clinical benefit of nonstatin medications, such as niacin, among patients with CKD. Both gemfibrozil and fenofibrate are renally cleared so they either require dose reduction or should be avoided depending on the severity of CKD. The dyslipidemia of CKD is characterized primarily by hypertriglyceridemia and reduced levels of high-density lipoprotein cholesterol. Therefore, therapies targeting triglyceride reduction may modify cardiovascular risk. Three studies have examined the relationship between marine derived n-3 fatty acids and cardiovascular outcomes; however, all were performed in patients on hemodialysis and used a less tailored n-3 fatty acid formulation than icosapent ethyl.^29–31^ Thus, there remains a critical need to test therapies for hypertriglyceridemia and related cardioprotection and ascertain their efficacy and safety among patients across the broad range of kidney disease.

Formal statistical testing did not demonstrate heterogeneity for the primary and key secondary composite end points with respect to baseline renal function, and therefore, the overall results of the REDUCE-IT study apply to the entire study population. It is reassuring that the benefits of icosapent ethyl seen in the initial study manifest across eGFR categories, given that other commonly used cardiovascular medications may have less efficacy and greater adverse events among patients with CKD.

There are limitations to the present analysis. The REDUCE-IT study was not powered specifically for subgroup analyses. A creatinine clearance <30 mL·min–1 or the need for renal replacement therapy excluded patients from the REDUCE-IT study. Therefore, a small number of patients with severe CKD were enrolled in the study. Based on these enrollment numbers, the power to detect the potential benefits, safety, or risk of icosapent ethyl among this cohort was more limited. We performed analyses on the basis of prespecified subgroups and post hoc analyses, as well, that used eGFR staging categories. Last, urine samples were not collected routinely in REDUCE-IT. Therefore, microalbuminuria and other adverse events relying on specimen analysis may be underreported in both treatment arms.

Overall, the results of the REDUCE-IT RENAL analyses are consistent with the overall study results. Among statin-treated patients randomly assigned to icosapent ethyl 4 g daily (2 g twice daily), there were significant reductions in the primary and key secondary composite end points regardless of baseline eGFR. These benefits extend to significant reductions in myocardial infarction and cardiovascular death.

## Article Information

### Acknowledgments

The authors thank the Amarin team members who are not authors of this article but who contributed to the success of the trial and of these analyses, including K. Diffin, A. Granger, and G. Chester, for operational support; R. Bhavanthula, R.H. Iroudyassamy, J. Jin, D. Klevak, G. Liu, H. Panchal, J. Shi, R. Wang, and S.-R. Wang, for data management and statistical support; and K. Keating from Amarin and S. Mercuro from Brigham and Women’s Hospital for editorial assistance (limited to formatting and collation of coauthor comments); and the investigators, the study coordinators, and especially the patients who participated in REDUCE-IT (The Reduction of Cardiovascular Events with Icosapent Ethyl – Intervention Trial).

### Sources of Funding

The study was funded by Amarin Pharma, Inc., which was involved in the study design, collection, analysis, and interpretation of data, and development and review of this article. The decision to submit the article for publication was made by the authors.

### Disclosures

Dr Majithia reports receiving consulting fees from Abbott Vascular. Dr Bhatt serves as the Chair and International Principal Investigator for REDUCE-IT (Reduction of Cardiovascular Events with Icosapent Ethyl-Intervention Trial), with research funding from Amarin to Brigham and Women’s Hospital. Dr Bhatt also discloses the following relationships: Advisory Board: Cardax, CellProthera, Cereno Scientific, Elsevier Practice Update Cardiology, Janssen, Level Ex, Medscape Cardiology, MyoKardia, NirvaMed, Novo Nordisk, PhaseBio, PLx Pharma, Regado Biosciences; Board of Directors: Boston VA Research Institute, Society of Cardiovascular Patient Care, TobeSoft; Chair: Inaugural Chair, American Heart Association Quality Oversight Committee; Data Monitoring Committees: Baim Institute for Clinical Research (formerly Harvard Clinical Research Institute, for the PORTICO trial [Portico Re-sheathable Transcatheter Aortic Valve System US IDE Trial], funded by St. Jude Medical, now Abbott), Cleveland Clinic (including for the ExCEED trial [ExCEED: CENTERA THV System in Intermediate Risk Patients Who Have Symptomatic, Severe, Calcific, Aortic Stenosis], funded by Edwards), Contego Medical (Chair, PERFORMANCE 2 [Protection Against Emboli During Carotid Artery Stenting Using the Neuroguard IEP System]), Duke Clinical Research Institute, Mayo Clinic, Mount Sinai School of Medicine (for the ENVISAGE trial [Edoxaban Compared to Standard Care After Heart Valve Replacement Using a Catheter in Patients With Atrial Fibrillation], funded by Daiichi Sankyo), Population Health Research Institute; Honoraria: American College of Cardiology (Senior Associate Editor, *Clinical Trials and News*, ACC.org; Chair, ACC Accreditation Oversight Committee), Baim Institute for Clinical Research (formerly Harvard Clinical Research Institute; RE-DUAL PCI [Evaluation of Dual Therapy With Dabigatran vs. Triple Therapy With Warfarin in Patients With AF That Undergo a PCI With Stenting] clinical trial steering committee funded by Boehringer Ingelheim; AEGIS-II executive committee funded by CSL Behring), Belvoir Publications (Editor in Chief, *Harvard Heart Letter*), Canadian Medical and Surgical Knowledge Translation Research Group (clinical trial steering committees), Duke Clinical Research Institute (clinical trial steering committees, including for the PRONOUNCE trial [A Trial Comparing Cardiovascular Safety of Degarelix Versus Leuprolide in Patients With Advanced Prostate Cancer and Cardiovascular Disease], funded by Ferring Pharmaceuticals), HMP Global (Editor in Chief, *Journal of Invasive Cardiology*), *Journal of the American College of Cardiology* (Guest Editor; Associate Editor), K2P (Co-Chair, interdisciplinary curriculum), Level Ex, Medtelligence/ReachMD (CME steering committees), MJH Life Sciences, Population Health Research Institute (for the COMPASS [Cardiovascular Outcomes for People Using Anticoagulation Strategies] operations committee, publications committee, steering committee, and USA national coleader, funded by Bayer), Slack Publications (Chief Medical Editor, *Cardiology Today’s Intervention*), Society of Cardiovascular Patient Care (Secretary/Treasurer), WebMD (CME steering committees); Other: *Clinical Cardiology* (Deputy Editor), NCDR (National Cardiovascular Data Registry)-ACTION Registry Steering Committee (Chair), VA CART Research and Publications Committee (Chair); Research Funding: Abbott, Afimmune, Amarin, Amgen, AstraZeneca, Bayer, Boehringer Ingelheim, Bristol-Myers Squibb, Cardax, CellProthera, Cereno Scientific, Chiesi, CSL Behring, Eisai, Ethicon, Ferring Pharmaceuticals, Forest Laboratories, Fractyl, Garmin, HLS Therapeutics, Idorsia, Ironwood, Ischemix, Janssen, Lexicon, Lilly, Medtronic, MyoKardia, NirvaMed, Novartis, Novo Nordisk, Owkin, Pfizer, PhaseBio, PLx Pharma, Regeneron, Roche, Sanofi, Synaptic, The Medicines Company, 89Bio; Royalties: Elsevier (Editor, *Cardiovascular Intervention: A Companion to Braunwald’s Heart Disease*); Site Co-Investigator: Abbott, Biotronik, Boston Scientific, CSI, St. Jude Medical (now Abbott), Philips, Svelte; Trustee: American College of Cardiology; Unfunded Research: FlowCo, Merck, Takeda. Dr Miller reports receiving consulting fees from Amarin and Akcea. Dr Brinton reports receiving fees as a speaker from Amarin, Amgen, Kowa, Regeneron, and Sanofi-Aventis, and consulting fees from Akcea, Amarin, Amgen, Esperion, Kowa, Medicure, PTS Diagnostics, Regeneron, and Sanofi-Aventis. Dr Jacobson reports receiving consulting fees from Amgen, Esperion, Novartis, Regeneron, and Sanofi. Dr Steg reports receiving research grant funding from Amarin, Bayer, Merck, Sanofi, and Servier; and speaking or consulting fees from Amarin, Amgen, AstraZeneca, Bayer/Janssen, Boehringer-Ingelheim, Bristol-Myers Squibb, Idorsia, Lilly, Merck, Novartis, Novo-Nordisk, Pfizer, Regeneron, Sanofi, and Servier. Dr Ketchum, R.T. Doyle, and Drs Juliano, Jiao, and Granowitz report being employed by and being stock shareholders of Amarin Pharma. Dr Tardif reports receiving grant support from AstraZeneca, Esperion, and Ionis, grant support and consulting fees from DalCor, grant support and fees for serving as cochairman of an executive committee from Pfizer, grant support and fees for serving on an executive committee from Sanofi, and grant support and consulting fees from Servier and holding a minor equity interest in DalCor and a patent (US 9,909,178 B2) on Dalcetrapib for Therapeutic Use. Dr Budoff has received grant support and is on the speaker’s bureau for Amarin Pharmaceuticals. Dr Ballantyne reports receiving consulting fees from Arrowhead, AstraZeneca, Eli Lilly, Matinas BioPharma, Merck, Boehringer Ingelheim, Novo Nordisk, Denka Seiken, and Gilead and grant support (paid to his institution) and consulting fees from Amarin, Amgen, Esperion, Novartis, Regeneron, Sanofi-Synthelabo, and Akcea. Dr Friedman serves on the Scientific advisory board of GI Dynamics, receives consulting fees from Goldfinch Bio, and is a Council member of the International Society of Renal Nutrition and Metabolism. No other potential conflict of interest relevant to this article was reported.

### Supplemental Material

Table S1

Figures S1–S7

## Supplementary Material


